# Clinical definition of secondary resistance to immunotherapy in non‐small cell lung cancer

**DOI:** 10.1111/1759-7714.15157

**Published:** 2023-11-14

**Authors:** Dingzhi Huang, Gen Lin, Qian Chu, Yi Hu, Jun Wang, Zhijie Wang, Fan Yang, Wenzhao Zhong, Chengzhi Zhou, Bo Zhu, Xinghao Ai, Baoshan Cao, Yabing Cao, Mingqiu Chen, Xiaohui Chen, Tianqing Chu, Jianchun Duan, Yun Fan, Yong Fang, Shuitu Feng, Weineng Feng, Hui Guo, Chengbo Han, Yong He, Shaodong Hong, Jie Hu, Meijuan Huang, Yan Huang, Da Jiang, Kan Jiang, Richeng Jiang, Bo Jin, Shi Jin, Jisheng Li, Min Li, Ziming Li, Chao Li, Jie Lin, Anwen Liu, Si‐Yang Maggie Liu, Yutao Liu, Zhefeng Liu, Zhe Liu, Zhenhua Liu, Zhentian Liu, Zhigang Liu, Yuping Lu, Tangfeng Lv, Zhiyong Ma, Qian Miao, Min Peng, Xingxiang Pu, Xiu Bao Ren, Jianzhen Shan, Jinlu Shan, Peng Shen, Bo Shen, Meiqi Shi, Yong Song, Zhengbo Song, ChunXia Su, Jianguo Sun, Panwen Tian, Jinliang Wang, Feng Wang, Huijuan Wang, Jialei Wang, Qian Wang, Wenxian Wang, Yan Wang, Lin Wu, Fang Wu, Yang Xia, Congying Xie, Conghua Xie, Tao Xin, Jianping Xiong, Haipeng Xu, Song Xu, Yiquan Xu, Bin Xu, Chunwei Xu, Xiaolong Yan, Zhenzhou Yang, Wenxiu Yao, Yao Yu, Ye Feng, Zongyang Yu, Yongfeng Yu, Dongsheng Yue, Haibo Zhang, HongMei Zhang, Li Zhang, Longfeng Zhang, Qiuyu Zhang, Tongmei Zhang, Bicheng Zhang, Jun Zhao, Mingfang Zhao, Xiaobin Zheng, Fengqiao Zhong, Jin Zhou, Penghui Zhou, Zhengfei Zhu, Juntao Zou, Zihua Zou

**Affiliations:** ^1^ Department of Thoracic Oncology Tianjin Medical University Cancer Institute and Hospital Tianjin People's Republic of China; ^2^ Department of Thoracic Oncology Clinical Oncology School of Fujian Medical University, Fujian Cancer Hospital Fuzhou People's Republic of China; ^3^ Department of Oncology, Tongji Hospital, Tongji Medical College Huazhong University of Science and Technology Wuhan People's Republic of China; ^4^ Senior Department of Oncology Chinese PLA General Hospital Beijing People's Republic of China; ^5^ Department of Oncology The First Affiliated Hospital of Shandong First Medical University & Shandong Provincial Qianfoshan Hospital Ji'nan People's Republic of China; ^6^ Department of Medical Oncology, National Cancer Center/National Clinical Research Center for Cancer/Cancer Hospital Chinese Academy of Medical Sciences and Peking Union Medical College Beijing People's Republic of China; ^7^ Department of Thoracic Surgery Peking University People Hospital Beijing People's Republic of China; ^8^ Guangdong Lung Cancer Institute, Guangdong Provincial People's Hospital Guangdong Academy of Medical Sciences Guangzhou People's Republic of China; ^9^ Pulmonary and Critical Care Medicine, Guangzhou Institute of Respiratory Health, National Clinical Research Center for Respiratory Disease, National Center for Respiratory Medicine, State Key Laboratory of Respiratory Diseases The First Affiliated Hospital of Guangzhou Medical University Guangzhou People's Republic of China; ^10^ Institute of Cancer, Xinqiao Hospital Army Medical University Chongqing People's Republic of China; ^11^ Shanghai Lung Cancer Center, Shanghai Chest Hospital Shanghai Jiao Tong University School of Medicine Shanghai People's Republic of China; ^12^ Cancer center Peking University Third Hospital/ Department of medical oncology and radiation sickness, Peking University Third Hospital Beijing People's Republic of China; ^13^ Department of oncology Kiang Wu Hospital Macau People's Republic of China; ^14^ Department of Thoracic Radiation Oncology, Clinical Oncology School of Fujian Medical University Fujian Cancer Hospital Fuzhou People's Republic of China; ^15^ Department of Thoracic Surgery, Clinical Oncology School of Fujian Medical University Fujian Cancer Hospital Fuzhou People's Republic of China; ^16^ Respiratory Department, Shanghai Chest Hospital Shanghai Jiao Tong University School of Medicine Shanghai People's Republic of China; ^17^ Department of Medical Oncology Zhejiang Cancer Hospital Hangzhou People's Republic of China; ^18^ Department of Medical Oncology, Sir Run Run Shaw Hospital Zhenjiang University School of Medicine Hangzhou People's Republic of China; ^19^ Department of Medical Oncology Fudan University Shanghai Cancer Center Xiamen Hospital Xiamen People's Republic of China; ^20^ Department of Pulmonary Oncology The First People's Hospital of Foshan Foshan People's Republic of China; ^21^ Department of Medical Oncology The First Affiliated Hospital of Xi'an Jiaotong University Xi'an People's Republic of China; ^22^ Department of Oncology Shengjing Hospital of China Medical University Shenyang People's Republic of China; ^23^ Department of Respiratory Medicine, Xinqiao Hospital Army Medical University Chongqing People's Republic of China; ^24^ State Key Laboratory of Oncology in Southern China Sun Yat‐sen University Cancer Center Guangzhou People's Republic of China; ^25^ Zhongshan Hospital, Fudan University Shanghai Geriatric Center Shanghai People's Republic of China; ^26^ Division of Thoracic Tumor Multimodality Treatment and Department of Medical Oncology, Cancer Center, West China Hospital Sichuan University Chengdu People's Republic of China; ^27^ Department of Oncology The Fourth Affiliated Hospital of Hebei Medical University Shijiazhuang People's Republic of China; ^28^ Department of Medical Oncology The First affiliated hospital of China Medical University Shenyang People's Republic of China; ^29^ National Cancer Center/National Clinical Research Center for Cancer/Cancer Hospital &Shenzhen Hospital Chinese Academy of Medical Sciences and Perking Union Medical College Shenzhen People's Republic of China; ^30^ Department of Medical Oncology Qilu Hospital of Shandong University Ji'nan People's Republic of China; ^31^ Department of Respiratory Medicine, Xiangya Hospital Central South University Changsha People's Republic of China; ^32^ Department of Pathology Clinical Oncology School of Fujian Medical University, Fujian Cancer Hospital Fuzhou People's Republic of China; ^33^ Department of Medical Oncology The Second Affiliated Hospital of Kunming Medical University Kunming People's Republic of China; ^34^ Department of Medical Oncology The Second Affiliated Hospital of Nanchang University Nanchang People's Republic of China; ^35^ Department of Hematology, First Affiliated Hospital Ji'nan University Guangzhou People's Republic of China; ^36^ Department of Medical Oncology, Beijing Chest Hospital Capital Medical University Beijing People's Republic of China; ^37^ Department of Oncology, Shengli Clinical Medical College of Fujian Medical University Fujian Provincial Hospital Fuzhou People's Republic of China; ^38^ Department of Thoracic Oncology，Jiangxi Cancer Hospital Nanchang People's Republic of China; ^39^ Cancer Center The 10th Affiliated Hospital of Southern Medical University Dongguan People's Republic of China; ^40^ Department of Abdominal Oncology Clinical Oncology School of Fujian Medical University, Fujian Cancer Hospital Fuzhou People's Republic of China; ^41^ Department of Respiratory Medicine, Affiliated Jinling Hospital Medical School of Nanjing University Nanjing People's Republic of China; ^42^ Department of Respiratory Medicine Henan Cancer Hospital /Affiliated Cancer Hospital of Zhengzhou University Zhengzhou People's Republic of China; ^43^ Cancer center Renmin Hospital of Wuhan University Wuhan People's Republic of China; ^44^ Department of Thoracic Medical Oncology, Hunan Cancer Hospital/The Affiliated Cancer Hospital of Xiangya School of Medicine Central South University Changsha People's Republic of China; ^45^ Department of Biotherapy Tianjin Medical University Cancer Institute and Hospital Tianjin People's Republic of China; ^46^ Department of Medical Oncology The First Affiliated Hospital of Zhejiang University Zhejiang People's Republic of China; ^47^ Department of Medical Oncology, Daping Hospital Army Medical University Chongqing People's Republic of China; ^48^ Department of Oncology, Nanfang Hospital Southern Medical University Guangzhou People's Republic of China; ^49^ Department of Medical Oncology Jiangsu Cancer Hospital and Jiangsu Institute of Cancer Research and Affiliated Cancer Hospital of Nanjing Medical University Nanjing People's Republic of China; ^50^ Department of Clinical Trial Zhejiang Cancer Hospital Hangzhou People's Republic of China; ^51^ Department of Oncology, Shanghai Pulmonary Hospital & Thoracic Cancer Institute Tongji University School of Medicine Shanghai People's Republic of China; ^52^ Department of Pulmonary and Critical Care Medicine, Lung Cancer Center, West China Hospital Sichuan University, Precision Medicine Key Laboratory of Sichuan Province Chengdu People's Republic of China; ^53^ Department of Thoracic Medical Oncology Fudan University Shanghai Cancer Center Shanghai People's Republic of China; ^54^ Department of Respiratory Medicine, Affiliated Hospital of Nanjing University of Chinese Medicine Jiangsu Province Hospital of Chinese Medicine Nanjing People's Republic of China; ^55^ Department of Oncology, The Second Xiangya Hospital Central South University Changsha People's Republic of China; ^56^ Department of Respiratory and Critical Care Medicine Second Affiliated Hospital of Zhejiang University School of Medicine Hangzhou People's Republic of China; ^57^ Department of Radiation and Medical Oncology Second Affiliated Hospital of Wenzhou Medical University Wenzhou People's Republic of China; ^58^ Department of Pulmonary Oncology Zhongnan Hospital of Wuhan University Wuhan People's Republic of China; ^59^ Department of Oncology The Second Affiliated Hospital of Harbin Medical University Harbin People's Republic of China; ^60^ Department of Oncology The First Affiliated Hospital of Nanchang University Nanchang People's Republic of China; ^61^ Department of Lung Cancer Surgery Tianjin Medical University General Hospital Tianjin People's Republic of China; ^62^ Department of Thoracic Surgery, Tangdu Hospital Air Force Medical University Xi'an People's Republic of China; ^63^ Department of Cancer Center The Second Affiliated Hospital of Chongqing Medical University Chongqing People's Republic of China; ^64^ Department of Medical Oncology, Sichuan Cancer Hospital University of Electronic Science and Technology of China Chengdu People's Republic of China; ^65^ Department of Medical Oncology, Xiamen Key Laboratory of Antitumor Drug Transformation Research The First Affiliated Hospital of Xiamen University, School of Medicine, Xiamen University Xiamen People's Republic of China; ^66^ Department of Respiratory Medicine, The 900th Hospital of the Joint Logistic Support Force People's Liberation Army of China Fuzhou People's Republic of China; ^67^ Department of Lung Cancer Tianjin Medical University Cancer Institute and Hospital Tianjin People's Republic of China; ^68^ Department of Oncology Guangdong Provincial Hospital of Chinese Medicine Guangzhou People's Republic of China; ^69^ Department of Clinical Oncology, Xijing Hospital Air Force Medical University Xi'an People's Republic of China; ^70^ Institute of Immunotherapy Fujian Medical University Fuzhou People's Republic of China; ^71^ Key Laboratory of Carcinogenesis and Translational Research(Ministry of Education/Beijing), Department I of Thoracic Oncology Peking University Cancer Hospital and Institute Beijing People's Republic of China; ^72^ Department of Radiation Oncology Fudan University Shanghai Cancer Center Shanghai People's Republic of China; ^73^ Department of Respiratory Medicine The First Affiliated Hospital of Nanchang University Nanchang People's Republic of China

**Keywords:** immunotherapy, NSCLC, secondary resistance

## Abstract

Immune checkpoint inhibitors (PD‐1/PD‐L1 and CTLA‐4 blockade) have revolutionized the treatment landscape in non‐small cell lung cancer (NSCLC). Secondary resistance to immunotherapy (IO), which poses a substantial challenge in clinical settings, occurs in several initial responders. Currently, new treatment approaches have been extensively evaluated in investigational studies for these patients to tackle this difficult problem; however, the lack of consistency in clinical definition, uniform criteria for enrollment in clinical trials, and interpretation of results remain significant hurdles to progress. Thus, our expert panel comprehensively synthesized data from current studies to propose a practical clinical definition of secondary resistance to immunotherapy in NSCLC in metastatic and neoadjuvant settings. In addition to patients who received IO alone (including IO‐IO combinations), we also generated a definition for patients treated with chemotherapy plus IO. This consensus aimed to provide guidance for clinical trial design and facilitate future discussions with investigators. It should be noted that additional updates in this consensus are required when new data is available.

## INTRODUCTION

Immunotherapies, especially PD‐1/PD‐L1 and CTLA‐4 blockade, have revolutionized the treatment paradigm for non‐small cell lung cancer (NSCLC) and become the standard treatment for the early and advanced stages of NSCLC without driving oncogenic mutation.^1^, ^2^, ^3^, ^4^, ^5^, ^6^, ^7^ Nevertheless, secondary resistance (or acquired resistance) still occurs in a large proportion of initial responders (i.e., disease progression following the initial benefit of immunotherapy), which poses a significant challenge for clinicians. Several clinical trials^8^, ^9^, ^10^, ^11^ (new agents or a combination of available treatment options) are currently under investigation to provide effective strategies to overcome this hurdle. However, none of these methods has yielded promising results. Undoubtedly, there is a lack of uniformity in the enrollment criteria in these trials; consequently, the activity of some specific therapies could be obscured by the undifferentiated definition of resistance. Taking primary and secondary resistance as an example, which may be fundamentally distinct,^12^, ^13^ tumors with secondary resistance to immunotherapy may still have antitumor immunity that could be reinvigorated with additional therapies. In contrast, primary resistance may be intrinsically refractory to immunomodulation. Therefore, a clear definition of secondary resistance to immunotherapy in NSCLC is beneficial for standardizing the enrollment criteria and keeping data collection consistent with data analysis, which ultimately facilitates therapeutic breakthroughs.

Previous articles published in the JITC and AO^14^, ^15^ have facilitated a broad discussion on the clinical definition of resistance to immunotherapy. Based on more reliable data, our expert panel established a pragmatic consensus that accurately reflects clinical scenarios to encourage clinicians to develop a clearer understanding of acquired resistance to immunotherapy. However, it should be noted that some controversies remain in our consensus (our panel members did not agree on every question), and we will update this consensus as more data becomes available.

In this consensus, we mainly propose the clinical definition of secondary resistance to immunotherapy in metastatic and neoadjuvant settings. In addition to immunotherapy (IO), the acquired resistance to chemotherapy is also defined in the following discussion, which is the most significant difference from previous articles. In contrast, adjuvant immunotherapy and consolidated immunotherapy for locally advanced NSCLC are excluded because no observation parameter is available to dynamically and practically evaluate the efficacy of adjuvant immunotherapy. Although some studies have demonstrated the significant potential of MRD in adjuvant settings,^16^, ^17^, ^18^, ^19^ additional data are needed. The efficacy of consolidated immunotherapy is also easily affected by radiation and chemotherapy in locally advanced NSCLC.

## CLINICAL DEFINITION OF SECONDARY RESISTANCE TO IMMUNOTHERAPY ALONE IN THE METASTATIC SETTING

### Cases in patients who experience disease progression following CR/PR or SD ≥6 months could be defined as secondary resistance

Given that the resistance mechanism to CTLA‐4 blockade could overlap with PD‐1/PD‐L1 inhibition, we suggested including patients treated with PD‐(L)1 plus CTLA‐4 checkpoint inhibition and those treated with PD‐(L)1 monotherapy in the definition of secondary resistance. Our expert panel recommended that patients who achieved complete response/radiological responders (CR/PR) (no duration of response threshold is required) or SD≥6 months could be defined as beneficiaries of immunotherapy. Multiple studies have indicated that patients who achieved CR/PR during immunotherapy treatment would experience substantial survival benefits. Notably, significantly higher survival rates were observed in patients who demonstrated a radiological response (4‐year OS rate > 60%) at the six‐month of nivolumab treatment than their counterparts (SD/PD, 4‐year Overall survival (OS) rate < 30%), as shown in a pooled analysis from CheckMate 017/057/063/003^20^ studies. Another pooled analysis from the CheckMate 012/817/568/227^21^ studies also revealed a remarkable survival advantage for responders (CR/PR) administered with nivolumab plus ipilimumab (42‐month OS rate over 60%, which was lower than 30% for SD/PD patients). Furthermore, in the KEYNOTE‐024^4^ and 042^22^ studies, a higher proportion of radiological responders (CR/PR) received full cycles of pembrolizumab (024:45%, 042:49.4%, while this number was only 5% for SD patients). In addition, previous results from KEYNOTE‐024^4^ and CheckMate 017/057^23^, ^24^ studies demonstrated that CR/PR patients were much less likely to develop progression in the short term (the probability of disease progression at 3‐ and 6‐months was 0% and 10%, respectively); therefore, we did not set the duration of the response threshold for radiological responders in this definition of acquired resistance.

However, whether patients with stable diseases can benefit from immunotherapy remains controversial because of limited data. “Stable disease” represents a heterogeneous mix of clinically nonresponding patients, those with indolent disease, and some patients with true benefit to immunotherapy. Since this definition applies only to metastatic NSCLC, tumors with indolent growth patterns can hardly be observed in this scenario. Meanwhile, previous data indicated that the SD population was correlated with a moderate survival advantage compared with the PD population. Additionally, from a clinical perspective, the duration of stable disease longer than 6 months is not inferior to the median Progression‐free survival (PFS) of platinum‐doublet chemotherapy; therefore, our panel recommended that patients who achieved SD≥6 months could also be regarded as beneficiaries of immunotherapy. New tools such as imaging techniques, ctDNA, and immune cell dynamics^25^, ^26^, ^27^, ^28^ may help identify true responders in the SD population and should be further investigated.

Considering the relatively low false positive rate for defining disease progression based on a single scan, particularly for patients who achieved CR/PR, we do not routinely recommend confirmatory scans in clinical practice. However, validating imaging or repeated biopsy should be considered if patients experience progression in oligo‐ or solitary lesions, especially in the lung or lymph nodes, to exclude pseudo‐progression^29^, ^30^, ^31^ and immune checkpoint inhibitor‐induced sarcoidosis‐like granulomas^32^, ^33^ (which are both rare (<5%) during immunotherapy in NSCLC). In addition, confirmatory imaging may be necessary for patients with progressive disease who have improved clinical symptoms or performance status.

## CLINICAL DEFINITION OF SECONDARY RESISTANCE TO IMMUNOTHERAPY PLUS CHEMOTHERAPY IN THE METASTATIC SETTING

### Cases in patients who develop disease progression with PFS≥18 months (first‐line setting) or PFS≥12 months (later‐line setting) could be defined as secondary resistance

Previous articles published in JITC and AO excluded patients treated with a combination of chemotherapy plus IO because of the difficulty in determining which component (or both) is/are the active agent(s) of response and ascribing acquired resistance to a specific agent. Notably, there is a significant unmet need in clinical practice if we establish the definition of secondary resistance to immunotherapy alone because chemotherapy plus IO is the mainstream treatment option for metastatic NSCLC, especially in patients with PD‐L1 < 50%.

Hence, we attempted to define this scenario in our consensus. Undoubtedly, the number of patients treated with chemotherapy without disease progression would decrease sharply over time. However, this is not the case for immunotherapy; that is, the proportion of patients without progression would remain relatively stable as time lengthens, and the longer the time, the fewer the progression events (plateau effect). Therefore, it is necessary to identify a cutoff point at which patients in the chemotherapy group barely experience stable disease (5% is statistically deemed as the threshold for a small probability event that is unlikely to occur in most cases) so that we could theoretically ascribe the survival benefit most likely to IO rather than chemotherapy.

As demonstrated in previous studies conducted over the last 20 years, only around 5% of patients treated with first‐line chemotherapy would experience progression‐free survival at 18 months of treatment; however, this proportion could rise to 25%–40% for chemo plus IO regimen^2^, ^3^, ^34^, ^35^, ^36^, ^37^, ^38^, ^39^, ^40^, ^41^, ^42^, ^43^, ^44^, ^45^, ^46^; Similarly, chemotherapy alone in a later‐line setting could only yield around 5% progression‐free survival at 12‐months of treatment.^23^, ^24^, ^47^, ^48^, ^49^ Based on these data, our expert panel suggested that it is reasonable to set the cutoff value of PFS at 18 months (first‐line setting) and 12 months (later‐line setting). Notably, several patients who genuinely benefit from immunotherapy would be omitted from this definition; meanwhile, patients who present a durable response to chemotherapy rather than IO could also be included in this scenario. Although this definition is imperfect, establishing an appropriate cutoff value could help clinicians roughly screen responders more accurately. The recommendations for confirmatory scans were the same as above.

## CLINICAL DEFINITION OF SECONDARY RESISTANCE IN PATIENTS (METASTATIC NSCLC) WHO DEVELOP PROGRESSION FOLLOWING THE COMPLETION OF IO TREATMENT

### Disease progression occurs within 6 months of completion of immunotherapy could be defined as secondary resistance; otherwise, retreatment of immunotherapy is required

Generally, the receptor occupancy of PD‐1/PD‐L1 inhibitors begins to decline 2–3 months after the last drug dose^50^ and is usually not identifiable at 6 months. Nevertheless, memory T cells demonstrate antitumor effects without receptor occupancy. Given the complicated antitumor immunity process, confirming the retreatment timing based only on pharmacokinetics is inadequate. Thus, we searched for a solution from a clinical perspective because it was difficult to identify the timing of rechallenge at the mechanism level.

A previous article published in AO that reviewed retreatment data from the KEYNOTE‐010^51^ and NCT01693562 trials^52^ showed that the majority of retreatment responses occurred after a six‐month treatment‐free interval (15/43 responses if retreatment interval >6 months vs. 1/18 responses if retreatment interval <6 months, Fisher's *p* = 0.02; therefore, patients could barely benefit from IO rechallenge if the retreatment interval was <6 months). Another pooled analysis of KEYNOTE‐024/042/598^53^ studies revealed that the ORR for pembrolizumab rechallenge was 19.3% (11/57), and all responders had retreatment intervals of >6 months. Therefore, previous data suggested that for patients with progressive events after long treatment‐free intervals, it is necessary to determine whether this represents true acquired resistance or could be sensitive to PD‐1/PD‐L1 inhibition. For these reasons, our expert panel recommended that it is reasonable to establish a treatment‐free interval of 6 months as the timing of rechallenge. The recommendations for confirmatory scans were the same as above.

## CLINICAL DEFINITION OF SECONDARY RESISTANCE TO IMMUNOTHERAPY ALONE IN A NEOADJUVANT SETTING

### For patients who experience radiological CR/PR or pCR/MPR (whether they receive adjuvant IO or not, disease progression occurs within 6 months after their last dose of checkpoint inhibitor) could be defined as secondary resistance; otherwise, retreatment of immunotherapy is required

Patients who achieve CR/PR in neoadjuvant settings could benefit from immunotherapy, similar to those with metastatic diseases. Given that the radiological response is not fully parallel to the pathological response^54^, ^55^ (prior data have shown that patients with the radiologically stable disease could also experience pathological complete response [pCR/MPR]), the pathological response should also be included as a parameter to evaluate whether patients could benefit from neoadjuvant immunotherapy. In this section, we reached a consensus on defining a patient who could benefit from IO treatment. However, it is rather difficult for us to identify the disease‐free interval as the timing of rechallenge based on the available data. More specifically, if a patient is defined as a beneficiary of neoadjuvant immunotherapy, it is appropriate to define secondary resistance when recurrence occurs during adjuvant immunotherapy; however, it remains a substantial challenge to confirm an appropriate disease‐free interval for a patient whose progression occurs following the cessation of immunotherapy. Given that patients who develop disease progression after the termination of adjuvant targeted therapy could still respond to retreatment,^56^, ^57^ this may also be the case for perioperative immunotherapy; therefore, after long disease‐free intervals, it is necessary to determine whether this represents acquired resistance or could be sensitive to PD‐1/PD‐L1 inhibition. Unfortunately, data on the retreatment of immunotherapy in perioperative settings are scarce, and we could not perform an exhaustive analysis to reach an agreement. Hence, we could only make a preliminary recommendation to set a disease‐free interval of 6 months after the last dose of checkpoint inhibitor as the timing of rechallenge. Additional updates are required when new data are available. Finally, it should be noted that a biopsy must be performed to confirm recurrence.

## CLINICAL DEFINITION OF SECONDARY RESISTANCE TO CHEMOTHERAPY PLUS IMMUNOTHERAPY IN A NEOADJUVANT SETTING

### For patients who experience pCR/MPR, whether they receive adjuvant IO or not, disease progression occurs within 6 months after their last dose of checkpoint inhibitor could be defined as secondary resistance; otherwise, retreatment of immunotherapy is required

Similar to the metastatic setting, there is a significant unmet need in clinical practice if we only discuss immunomonotherapy in view of chemotherapy plus IO being the mainstream treatment option in neoadjuvant settings. In particular, we were unable to determine which component (or both) is/are the active agent(s) responsible for the response and to ascribe acquired resistance to a specific agent for this combination regimen using current techniques; therefore, we could only roughly screen out responders from a clinical perspective. Furthermore, because of the confounding effects of chemotherapy (ORR for neoadjuvant chemotherapy is 30%–40%), it is unreasonable to apply radiological evaluation as a parameter to identify which patient could benefit from immunotherapy under combination treatment. However, the interference of chemotherapy could be largely excluded by the use of pathological response because pCR and MPR for neoadjuvant chemotherapy were generally lower than 5% and 10%. In contrast, chemo plus IO demonstrated much higher pCR (20%–40%, odds ratio 5–10) and MPR (35%–55%, odds ratio 5–10).^6^, ^58^, ^59^ Hence, we could theoretically ascribe the contribution more likely to IO than to chemotherapy if patients achieved pCR/MPR. Similarly, a disease‐free interval of 6 months after the last dose of checkpoint inhibitor was proposed as the timing of rechallenge in this setting.

In conclusion, we propose a practical clinical definition of secondary resistance to immunotherapy in NSCLC in two distinct scenarios, metastatic and neoadjuvant settings, based on data from current studies. In addition to patients who received IO alone (including IO‐IO combinations), we also generated a definition for patients treated with chemotherapy plus IO. This consensus aimed to provide guidance for clinical trial design and facilitate future discussions with investigators. Additional updates to this consensus are required when new data become available.

## AUTHOR CONTRIBUTIONS

Dingzhi Huang, Gen Lin, Qian Chu, Yi Hu, Jun Wang, Zhijie Wang, Fan Yang, Wenzhao Zhong, Chengzhi Zhou and Bo Zhu were responsible for the design of this article. Zihua Zou, Dingzhi Huang, Gen Lin drafted this manuscript. All authors reviewed the final version of manuscript.

## FUNDING INFORMATION

None.

## CONFLICT OF INTEREST STATEMENT

The authors report no conflicts of interest related to this study.

## Supporting information


**DATA S1.** Supporting Information.
