# Supplementary material for: Clinical definition of secondary resistance to immunotherapy in non‐small cell lung cancer
Source: Thorac Cancer. 2023 Nov 14;14(34):3421–9. doi: 10.1111/1759-7714.15157 (PMC10693946; doi:10.1111/1759-7714.15157)
Supplement: Supplementary file 1 — DATA S1. Supporting Information. [file TCA-14-3421-s001.docx]

**147 experts in total participated in this voting section**

**Question 1:**

**Cases in patients with metastatic NSCLC treated with IO alone who experience disease progression following CR/PR or SD≥6 months could be defined as secondary resistance.**

**A: agree (****98.55%)**

**B:****disagree (1.45%)**

**Question 2:**

**Cases in patients with metastatic NSCLC treated with IO plus chemotherapy who develop disease progression with PFS≥18 months (first-line setting) or PFS≥12 months (latter-line setting) could be defined as secondary resistance.**

**A: agree (****76.98%)**

**B:disagree (****23.02%)**

**Question 3:**

**For patients with metastatic NSCLC who completed the full course of IO treatment, disease progression occurs within six months of completion of IO could be defined as secondary resistance; otherwise, retreatment of IO is required.**

**A: agree (****84.89%)**

**B:disagree(****15.11%)**

**Question 4:**

**For patients treated with neoadjuvant IO alone, those who experience radiological CR/PR or pCR/MPR could be defined as beneficiaries of immunotherapy.**

**A: agree (****94.2%)**

**B:disagree(****5.8%)**

***In perioperative setting, after long disease-free intervals, it is necessary to determine whether this represents acquired resistance or could be sensitive to PD-1/PD-L1 inhibition. However, we did not reach a consensus on the disease-free interval as the timing of rechallenge (no requirement or 3 or 6 or 12 months for disease-free interval were proposed by different experts).**

**Question 5:**

**For patients treated with neoadjuvant chemotherapy plus IO, those who experience pCR/MPR could be defined as beneficiaries of immunotherapy.**

**A: agree (****73.19%)**

**B:disagree(****26.81%)**

***In perioperative setting, after long disease-free intervals, it is necessary to determine whether this represents acquired resistance or could be sensitive to PD-1/PD-L1 inhibition. However, we did not reach a consensus on the disease-free interval as the timing of rechallenge (no requirement or 3 or 6 or 12 months for disease-free interval were proposed by different experts).**
